# Exploring the directionality in the relationship between descriptive and injunctive parental and peer norms and snacking behavior in a three-year-cross-lagged study

**DOI:** 10.1186/s12966-020-00977-w

**Published:** 2020-06-15

**Authors:** K. E. Bevelander, W. J. Burk, C. R. Smit, T. J. van Woudenberg, L. Buijs, M. Buijzen

**Affiliations:** 1 Communication Science, Behavioural Science Institute, Radboud University, P.O. Box 9104, 6500 HE Nijmegen, the Netherlands; 2 Primary and Community Care, Radboud Institute for Health Sciences, Radboud University and Medical Centre, Nijmegen, the Netherlands; 3 Erasmus School of Social and Behavioural Sciences, Erasmus University Rotterdam, Rotterdam, the Netherlands

**Keywords:** Descriptive norms, Injunctive norms, Parental influence, Peer influence, Adolescents, (un)healthy snack food intake, Cross-lagged panel model, Bidirectional relationship

## Abstract

**Background:**

People’s eating behavior is assumed to be influenced by what other people do (perceived *descriptive norms*) and what others approve of (perceived *injunctive norms*). It has been suggested that adolescents are more susceptible to peer norms than parental norms, because they experience a strong need for group acceptance that leads to conforming to peer group norms. The current study examined changes in snacking behavior and four types of social norms (i.e., parental and peer descriptive and injunctive norms) that promoted fruit and vegetable intake among adolescents. This study was the first to examine whether snacking behavior also influenced norm perceptions by testing the directionality of these associations.

**Methods:**

The study consisted of 819 participants (*M* [SD] age = 11.19 [1.36]; 46.1% boys), collected at three time points (T1 = 2016, T2 = 2017 and T3 = 2018) during the *MyMovez* project. Self-reported frequency of snack consumption, perceived parental and peer descriptive and injunctive norms were assessed. The primary analysis consisted of a series of cross-lagged autoregressive models specified in a structural equation modeling framework.

**Results:**

Model comparisons testing the descriptive and injunctive norms in separate models and in an additional combined model revealed evidence for bi-directional associations between norms and snacking behavior. Descriptive peer and parent norms were not found to have an effect on subsequent snacking behaviors. Perceived injunctive parental norms were positively associated with healthy snack food intake and negatively associated with unhealthy snack intake (*forward* direction). Injunctive peer norms were negatively associated with healthy snack food intake. In addition, higher unhealthy snack food intake was negatively associated with the perception of descriptive and injunctive parental norms 1 year later (*reversed* direction). We did not find peer norms to be more closely associated with changes in snacking behaviors compared to parental norms.

**Conclusions:**

Parents expecting their children to snack healthy had a positive influence on healthy snacking behavior whereas only acting as a healthy role model did not. Future research should address the possible interaction between descriptive and injunctive norms. Research should also take into account the bi-directional relations between eating behaviors and normative perceptions.

## Introduction

The prevalence of overweight and obesity is still rising among school-aged children in Western countries [1, 2]. Given that eating habits persist in adulthood, the importance of establishing healthy eating practices during childhood, and maintaining them throughout adolescence, has been acknowledged for a long time [3, 4]. Eating a sufficient amount of fruits and vegetables is an important factor in preventing obesity and avoiding chronic diseases [5, 6]. Even so, most adolescents dislike fruit and vegetables and prefer high-fat and high-sugary ‘fast-food-style’ products [7, 8]. Research has shown that their (snack) food intake is strongly influenced by close social connections [9], which provides opportunities for prevention. The development of effective strategies to improve adolescents’ eating behaviors requires an understanding of how significant others (i.e., parents and peers) influence eating behavior [10]. In addition, it is important to examine whether adolescents’ own eating behavior influences their perception of what close others do and approve of. This study is the first study that explored this bi-directionality between parental and peer social norms and snack food intake among adolescents.

People tend to conform to other persons’ consumption behavior in various social contexts [11]. Specifically, empirical and cross-sectional studies have shown that people’s consumption behavior is influenced by *descriptive* and *injunctive* social norms. Perceived descriptive norms are informational non-coercive guidelines that people conform to, whereas injunctive norms exert pressure on one’s behavior and relate to the feeling of external expectations and (dis)approval of ‘(un)appropriate’ eating behavior [12]. These social norms have been identified as powerful mechanisms in determining adolescents’ and adults’ consumption behavior [11, 13–18]. Although the research field on social norms in adolescents is still evolving, few experimental normative studies have already shown promising findings to promote healthy consumption behavior [10]. For example, an intervention study successfully targeted water drinking among schoolchildren by influencing the peer group norm [19]. That is, selected peers were secretly instructed to promote water drinking by increasing their own water drinking behavior and talking about the benefits of water drinking to others, which increased water drinking and decreased the consumption of sugar-sweetened beverages among their class mates. Other experimental studies found that adolescents increased their vegetable intake after exposing them to information about the amount that their peers ate [20, 21]. In addition, an online study found that an injunctive peer norm promoting fruit and vegetable intake was associated with higher self-reported fruit and vegetable intake as well as lower unhealthy snack food intake [18]. These findings indicate that both descriptive and injunctive norms influence adolescents’ healthy consumption behavior. Notably, these perceived norms are typically assumed to affect subsequent eating behavior. The reversed impact of eating behaviors on perceived norms has yet to be empirically tested. Therefore, we also examined whether adolescents’ own eating behavior affected their perception of the social norm in a three-wave longitudinal study.

Although young people, and adolescents especially, are influenced by their peers’ consumption behavior [11, 16, 22], their parents also represent important figures in forming dietary patterns by serving as nutritional gatekeepers and role models [8, 23–26]. It has been suggested that parental influences decline when competing with peer influences, because adolescents experience a strong need for group acceptance that leads to conforming to normative behavior of peers [15, 16, 27–29]. Studies have found that even if adolescents have appropriate nutritional knowledge, they still choose fast-food-style products within their school and social environment because they think it is emotionally and socially too risky (e.g., ‘not cool’) to show interest in healthy eating [7, 16]. To our knowledge, only one cross-sectional study has examined both parental and peer descriptive and injunctive norms among adolescents. The study showed that only descriptive parental norms influenced self-reported fruit and vegetable consumption [30], which did not support literature suggesting that peer norms would be more important than parental norms. Notably, they also found negative correlations between parental as well as peer injunctive norms and healthy eating behavior, which could suggest potential ‘reactance effects’ [31]. Reactance effects were also found in previous studies on injunctive norms and eating behavior among adults, especially [32, 33]. It has been suggested that the pressure from an injunctive norm may lead to a dismissal or even backfiring of the intended effect, because people feel pressured or threatened in their sense of freedom [31].

Surprisingly, longitudinal research investigating the relative importance of parental and peer influences on eating behavior is scarce [34–37]. A study that examined adolescents’ intentions and self-reported fruit, vegetable and water consumption by integrating antecedents from different theoretical approaches (i.e., theory of planned behavior, social norms, and intrinsic motivation) in one model, found that perceived descriptive parental norms (and not injunctive nor any peer norms) predicted behavioral change on water drinking only [37]. Furthermore, a longitudinal study among a student population that focused merely on descriptive peer norms showed no evidence of descriptive norms being a reliable predictor of future snacking and drinking behavior. That is, perceptions about how much peers consumed sugary snack foods and (alcoholic) drinks had limited effect on students’ eating and drinking behavior 1 year later [35]. Altogether, more research is needed to determine the directional relationships between both parental and peer descriptive and injunctive norms on the promotion of healthy snacking behavior.

In this study, we not only examined the generally assumed ordering of norms preceding eating behavior (*forward*), but also considered the possibility of eating behaviors preceding perceived norms (*reversed*), as well as possible bi-directional associations (*reciprocal*) between these constructs [36]. For example, when we make assumptions about how much fruits and vegetables other people consume (i.e., descriptive norm), this perception could be biased by our own eating behavior. Notably, it also provides a different explanation to what is now assumed to be reactance to injunctive norms. It may be that when people usually do not eat a lot of fruits and vegetables - and are aware of this -, they assume and perceive others thinking that they should eat fruit and vegetables. So, one’s own eating behavior may, in fact, influence our perceptions of what others expect from us. In addition, social norms and snacking behavior may be related in a bi-directional manner in which the above mentioned processes are combined into positive and/or negative feedback loops.

The current study is the first to examine changes in snacking behavior and four types of social norms that promoted fruit and vegetable intake, and the directionality of these associations among school-aged adolescents by applying cross-lagged autoregressive models to a three-wave longitudinal study. To fully understand the potential impact of norms on snacking behavior and vice versa, we included fruit and vegetable (‘*core*’) snack food intake as well as sweet and savory (‘*non-core*’) snack food intake in our study [38, 39]. We took a step-wise approach by comparing four structural models to investigate the proposed cross-lagged effects for descriptive and injunctive norms separately. We had the following set of research questions (belonging to each step and model) to investigate the directionality between norms and behavior:

- RQ1. To what extent are social norms and snacking behaviors stable from T1 to T2, and T2 to T3 (i.e., *baseline* or *stability* model)?
- RQ2. Do parental and peer social norms at T1 predict snacking behaviors at T2, and from T2 to T3 (i.e., *forward* model)?
- RQ3. Do core and non-core snacking behaviors predict parental and peer social norms from T1 to T2, and T2 to T3 (i.e., *reversed* model)?
- RQ4. Are there bidirectional relationships between social norms and snacking behaviors (i.e., *reciprocal* model)?

## Methods

### Participants and procedure

Participants were recruited into a large-scale cross-sequential cohort study through their schools (21 primary (*n* = 453) and secondary (*n* = 500) schools) in the Netherlands as part of the so-called *MyMovez* project [40]. All (sub)urban schools following a regular education program were eligible for participation ranging from primary school level, and lower vocational training to secondary school. All schools located near the Nijmegen area were invited to participate in the *MyMovez* project. Further, Dutch Public Health Services (GGD) of each province were contacted to promote the *MyMovez* project at their regions. In addition, schools were invited to participate via personal contacts of students and researchers. This random procedure resulted in the participation of 21 (sub)urban schools throughout the Netherlands. Active written consent was obtained from the school directors, caretakers and the participants themselves. Participants received a smartphone with the *MyMovez* research application for nearly a week. They received random invitations to fill out questionnaires on their smartphone each day between 7:00 AM and 7:30 PM (but not during school hours, except for school breaks) (for detailed information about the recruitment procedure and the *Wearable Lab*, see [40]). Background and precursor variables were assessed during Phase I of the *MyMovez* project [40]. For the present study, we made use of data on frequency of snack consumption, perceived descriptive and injunctive parent and peer norms, and several background variables collected over a period of 3 years.

Data collection took place around February and March 2016 (T1), 2017 (T2) and 2018 (T3). The participants were allowed to enter and drop out when they wanted (also during a measurement period), because participation was voluntary. In addition, the participants attending the highest grade levels dropped out at T2 and T3, because they left school. From the 953 participants who initially had parental consent, the response rate was 71% at T1, 40% at T2 and 33% at T3. Participants who answered to the set of questions used for this study at least once were included in the study (*N* = 819; 47.5% primary school children; 46.1% boys; *M*(*SD*) age = 11.19 (1.36); > 90% Dutch origin).

### Measures

#### Core and non-core snack intake

Participant’s self-reported snack consumption was assessed by items from a food frequency questionnaire (FFQ) which accounted for Dutch food items based on the Dutch EPIC FFQ [41]. Participants were asked to recall every other day (i.e., three times per data wave on 2 weekdays and a weekend day)^1^ how many pieces of snack food items they consumed on the previous day with answering options ranging from 0 = *none* to 6 = *six or more*. Examples of frequently eaten snack food items were given after each question to further explain the items to the participants. The number of units were multiplied by the average kilo caloric value representing each food item category to place relative weight on the food items (e.g., a small cookie does not equal the energy value of a piece of pie).^2^ The participant’s reported consumption was averaged for each food item per wave. Next, a clear distinction was made between core and non-core items based on previous literature and nutritional guidelines of the Dutch Nutrition Centre [38, 39, 42]. This resulted in three snack food item categories: 1) core: fruit and vegetables; 2) non-core: small, large and wrapped cookies, sweet pastry, chocolates, chocolate bars, candy and liquorice, savory and warm (pastry) snacks, chips, ice cream, and 3) an in-between category: (skimmed) milk, cottage cheese, nuts. A proportional snack intake value was then calculated by adding up the three categories for a total snack consumption score and dividing the kilo caloric values for core and non-core snacks by the total snack consumption score. The two core and non-core measures were used in the analyses.

#### Descriptive norms

Perceived descriptive norms on fruit and vegetable snack intake were assessed with two separate items about parents and friends: ‘*How often do your parents/friends eat fruits and vegetables as a snack?*’ [30]. Response items ranged from 1 (‘*never’)* – 6 (‘*always*’)*.*

#### Injunctive norms

Participants’ perceptions of social pressure on their fruit and vegetable snack intake were assessed with two separate items for parents and friends: ‘*Do you think that your parents/friends believe you should eat fruits and vegetables as a snack?*’ [30]. Response options ranged from 1 (‘*no, certainly do not’*) – 6 (‘*yes, certainly do*’)*.*

#### Covariates

Age, sex and weight status were mentioned in empirical studies as potential confounders in the relationship between social norms and snack intake [11, 15, 43]. Demographic variables age and sex were supplied by the schools’ administration offices. Weight and length was measured each year by trained researchers following standard procedures. Standardized BMI scores were calculated accounting for variations in growth curves of children and adolescents [44].

### Strategy of analyses

Descriptive statistics were calculated to examine the distribution (minimum, maximum and means) of all model items and the differences between time points T1-T3. In addition, bivariate correlations among all model items were computed.

**Fig. 1:**
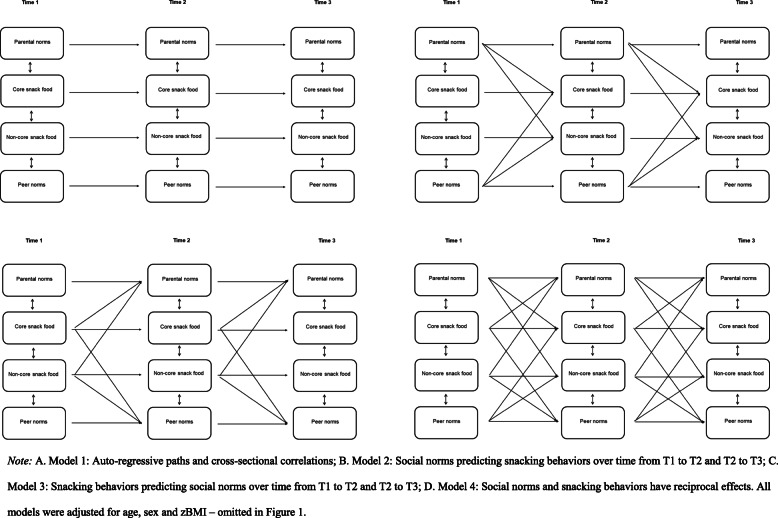
Baseline, forward, reversed and reciprocal models for social norms and snacking behavior

The primary analyses consisted of a series of competing cross-lagged autoregressive models specified in a structural equation modeling framework in Mplus version 7.2 [45, 46]. To examine our research questions, 2 sets of four models were compared for descriptive and injunctive norms on snack intake. Model 1 (answering RQ1) was the baseline or stability model, which only included autoregressive paths (estimating intra-individual stability) between the three assessments and all concurrent correlations among constructs. Model 2 (RQ2) examined social norms as predictors of snacking behaviors, and included the same parameters as Model 1, but also included cross-lagged paths from T1 and T2 social norms to T2 and T3 snack intake, respectively. Model 3 (RQ3) examined snacking behaviors as predictors of social norms, and included the same parameters as Model 1, but also included cross-lagged paths from T1 and T2 snack intake to T2 and T3 social norms, respectively. Model 4 (RQ4) examined bidirectional associations between social norms and snacking behaviors, and included all parameters specified in Models 1, 2 and 3. Figure 1 presents an overview of the four models. Age, sex, and BMI were included as covariates in the models on T1 based on their significant correlations with the model variables under investigation (see Table 2). The parameters in the models were estimated using (Full-Information) Maximum Likelihood estimation with robust standard errors (MLR in Mplus) to account for missing values and potential deviations from multivariate normality. In the additional analysis, we combined all variables (i.e., descriptive and injunctive parent and peer norms) in one model to examine the bi-directional relationship between norms and snacking behavior.

For each model, model fit information was assessed by the following fit indices: the χ^2^ test of model fit, the Standardized Root Mean Square Error of Approximation (RMSEA; satisfactory values below .06) [47], the Comparative Fit Index (CFI) and the Tucker-Lewis Index (cut-off values close to or above .90). Next, Chi-square difference tests with MLR scaling correction were assessed to assess which model(s) provided a significantly better fit to the data.

## Results

### Descriptive statistics

The distribution of study variables are presented in Table 1 and the correlations between all model variables including covariates are presented in Table 2. The difference tests displayed in Table 1 show that all study variables were stable over time, except for a statistically significant decrease in non-core snack intake between T1 and T2 only (*p* = .002). Table 2 shows cross-sectional correlations and there were significant positive correlations between descriptive parental norms and core snack intake across T1, T2 and T3, and negative correlations between descriptive parental norms and non-core food intake (all *p*-values <.05). Injunctive peer norms and non-core food intake were negatively correlated at T1 (*r* = −.09, *p* = .02) and T2 (*r* = −.13. *p* = .03). There were almost no significant correlations (all *p*-values >.05) between descriptive and injunctive peer norms and core snack intake (except for T2 injunctive peer norm and core snack intake (*r* = .13, *p* = .03).

**Table 1:** Distribution of study variables. MyMovez study, 2016, 2017 and 2018

	N	Min.	Max.	Mean	SD
**Time 1**					
Descriptive friend norm	678	1	6	3.54	1.13
Descriptive parent norm	678	1	6	4.01	1.31
Injunctive friend norm	615	1	6	3.29	1.64
Injunctive parent norm	615	1	6	4.75	1.45
Percentage core snack food (%)^a^	812	0	100	23.69	17.09
Percentage non-core snack food (%)^a^	812	0	100	63.55	20.85
**Time 2**					
Descriptive friend norm	389	1	6	3.59	1.10
Descriptive parent norm	389	1	6	4.05	1.18
Injunctive friend norm	285	1	6	3.34	1.56
Injunctive parent norm	289	1	6	4.58	1.39
Percentage core snack food (%)^a^	569	0	100	24.73	20.03
Percentage non-core snack food (%)^a^	569	0	100	59.79	23.73
**Time 3**					
Descriptive friend norm	310	1	6	3.44	1.02
Descriptive parent norm	311	1	6	3.90	1.18
Injunctive friend norm	315	1	6	3.18	1.57
Injunctive parent norm	315	0	6	4.70	1.31
Percentage core snack food (%)^a^	258	0	100	25.73	22.74
Percentage non-core snack food (%)^a^	258	0	100	61.55	28.54
	**Comparison T2-T1**^**b**^			**Comparison T3-T2**^**b**^	
	ΔMean	*P*-value^c^		ΔMean	*P*-value^c^
Descriptive friend norm	.05	n.s.		−.15	n.s.
Descriptive parent norm	.04	n.s.		−.15	n.s.
Injunctive friend norm	.05	n.s.		−.16	n.s.
Injunctive parent norm	−.17	n.s.		.12	n.s.
Percentage core snack food (%)^a^	1.04	n.s.		1	n.s.
Percentage non-core snack food (%)^a^	−3.76	.002		1.76	n.s.

^a^The percentage of core and non-core snack food intake from total (incl. Middle category) snack food intake is displayed in this table, but we used the proportion numbers (percentage divided by 100) in our analyses

^b^A positive difference value indicates higher values over time whereas a negative difference values reflects the opposite

^c^Wilcoxon Rank Compared Test

**Table 2:** Correlations between all model variables. MyMovez study, 2016, 2017 and 2018

	1	2	3	4	5	6	7	8	9	10	11	12	13	14	15	16	17	18	19	20	21
1- DN^a^ peer T1																					
2- DN^a^ peer T2	.19^**^																				
3- DN^a^ peer T3	.16^**^	.34^**^																			
4- DN^a^ parent T1	.44^**^	.11	.11																		
5- DN^a^ parent T2	.11	.50^**^	.15^**^	.37^**^																	
6- DN^a^ parent T3	.18^**^	.33^**^	.36^**^	.30^**^	.45^**^																
7- IN^b^ peer T1	.30^**^	.10	.01	.20^**^	..13^*^	.07															
8- IN^b^ peer T2	.19^**^	.27^**^	.24^**^	.11	.22^**^	.20^*^	.26^**^														
9- IN^b^ peer T3	.06	.09	.31^**^	.03	.07	.26^**^	.26^**^	.43^**^													
10- IN^b^ parent T1	.18^**^	.05	−.06	.32^**^	.17^**^	.07	.48^**^	.20^**^	.11												
11- IN^b^ parent T2	.06	.20^**^	.01	.13^*^	.35^**^	.31^**^	.09	.45^**^	.19^*^	.20^**^											
12- IN^b^ parent T3	.10	.10	.16^**^	.13	.18^**^	.39^**^	.17^*^	.21^*^	.38^**^	.28^**^	.34^**^										
13- Core snack T1	.07	.04	.07	.16^**^	.14^*^	.13^*^	.04	.09	.06	.15^**^	.03	.09									
14- Core snack T2	.07	.05	.06	.11^*^	.19^**^	.08	−.05	.13^*^	−.01	.15^**^	.17^**^	.10	.29^**^								
15- Core snack T3	.06	.02	.09	−.06	.09	.14^*^	.11	−.03	.02	.18^*^	.01	.05	.27^**^	.21^**^							
16- Non-core snack T1	−.12^**^	−.09	−.07	−.22^**^	−.21^**^	−.20^**^	−.09^*^	−.11	−.02	−.19^**^	−.10	−.10	−.79^**^	−.24^**^	−.27^**^						
17- Non-core snack T2	−.03	−.11^*^	−.13^*^	−.12^*^	−.23^**^	−.17^**^	−.01	−.13^*^	−.04	−.10	−.18^**^	−.03	−.20^**^	−.67^**^	−.22^**^	.26^**^					
18- Non-core snack T3	−.05	−.06	−.12	−.06	−.08	−.14^*^	−.11	−.02	−.03	−.14	−.04	−.01	−.31^**^	−.14^*^	−.73^**^	.36^**^	.27^**^				
19- Sex^c,d^	.07	−.03	.01	.08^*^	.14^**^	.03	.06	.03	.07	.03	.03	.01	.05	.02	.01	−.01	.02	.06			
20- Age T1	−.09^*^	−.17^**^	−.13^*^	−.11^**^	−.14^**^	−.08	−.02	−.04	−.08	.03	.00	.03	−.12^**^	−.12^**^	−.03	.13^**^	.10^*^	.01	.03		
21- zBMI T1	.05	.06	.15^*^	.05	−.06	.00	.00	−.01	.05	−.06	−.16^*^	−.09	.03	.05	−.09	−.08^*^	−.11^*^	−.04	−.03	−.04	

*Note.* T1 = Time 1; T2 = Time 2; T3 = Time 3; ^a^Descriptive norm; ^b^Injunctive norm; ^c^0 = boy and 1 = girl; ^d^Spearman’s rank correlation; Correlations are without missing value imputations. ^*^p < .05, ^**^p < .01

The bivariate correlations in Table 2 also showed that several descriptive and injunctive norms were correlated (e.g., descriptive peer norm with descriptive parental norm on T1, T2 and T3). Therefore, in the additional analysis, we explored whether combining the influence of all norms and snacking behavior in one model would lead to different findings than the planned main analyses.

### Main analyses

Table 3 provides an overview of goodness of fit indices for all four structural path models for descriptive and injunctive norms, separately. All models provided acceptable fit to the observed data with all Chi-Square test of model fits being significant (*p-*values < .01) and according to the values for RMSEA (.028–.032), CFI (.942–.978) and TLI (.942–.956).

**Table 3:** Goodness of fit statistics for the tested models on social norms and snacking behavior. MyMovez study (*N* = 819), 2016, 2017 and 2018

**Descriptive norm**	**Model 1 Baseline**^**a**^	**Model 2 Forward**^**b**^	**Model 3 Reversed**^**c**^	**Model 4 Reciprocal**^**d**^
RMSEA	.030	.032	.031	.033
CFI	.966	.970	.971	.973
TLI	.951	.946	.948	.942
AIC	5332.375	5337.657	5336.916	5341.779
Ssa BIC	5427.388	5444.930	5444.189	5461.312
*χ*^2^	111.730	101.645	100.343	89.839
*Df*	64	56	56	48
*P*	.0002	.0002	.0003	.0002
Scaling correction factor for MLR	1.1226	1.1285	1.1358	1.1446
**Chi-square difference test**				
Comparison with:	–	Model 1	Model 1	Model 1 / Model 2 / Model 3
Change in *χ*^2^		10.085	11.387	21.891 / 11.806 / 10.504
Change in *Df*		8	8	16 / 8 / 8
*P*		.271	.195	.164 / .174 / .167
**Injunctive norms**	**Model 1 Baseline**	**Model 2 Forward Causation**	**Model 3 Reversed Causation**	**Model 4 Reciprocal**
RMSEA	.029	.028	.030	.029
CFI	.970	.976	.971	.978
TLI	.952	.956	.948	.953
AIC	5683.262	5681.901	5688.783	5686.972
Ssa BIC	5778.200	5789.008	5795.970	5806.410
*χ*^2^	108.946	91.774	98.226	81.020
*Df*	64	56	56	48
*P*	.0004	.0018	.0004	.0020
Scaling correction factor for MLR	1.1005	1.1172	1.1139	1.1306
**Chi-square difference test**				
Comparison with:	–	Model 1	Model 1	Model 1 / Model 2 / Model 3
Change in *χ*^2^		17.172	10,72	27.926 / 10.754 / 17.206
Change in *Df*		8	8	16 / 8 / 8
*P*		.024	.237	.032 / .229 / .025
**Add. analyses Combined model**	**Model 1 Baseline**^**a**^	**Model 2 Forward**^**b**^	**Model 3 Reversed**^**c**^	**Model 4 Reciprocal**^**d**^
RMSEA	.034	.035	.036	.037
CFI	.937	.941	.940	.944
TLI	.905	.899	.897	.889
AIC	13,121.443	13,126.358	13,131.429	13,135.890
Ssa BIC	13,285.417	13,314.852	13,319.922	13,348.903
*χ*^2^	268.438	243.668	246.679	221.839
*Df*	136	120	120	104
*P*	.0000	.0000	.0000	.0000
Scaling correction factor for MLR	1.0704	1.0681	1.0756	1.0719
**Chi-square difference test**				
Comparison with:	–	Model 1	Model 1	Model 1 / Model 2 / Model 3
Change in *χ*^2^		24.77	21.759	46.599
Change in *Df*		16	16	32 / 16 / 16
*P*		.072	.166	.047 / .159 / .069

^a^Only auto-regressive effects and cross-sectional correlations

^b^Social norms predicting snack intake at T1 to T2, and T2 to T3, respectively

^c^Snack intake predicting social norms at T1 to T2, and T2 to T3, respectively

^d^Bi-directional relationships between social norms and snack intake

#### Descriptive norms

The chi-square difference tests between the models for descriptive norms showed that Models 2, 3 and 4 did not provide better insight than the baseline Model 1. This means that social norms and behaviors were stable over time (RQ1), and that there were no uni- or bi-directional relationships between parental and peer descriptive norms and snacking behavior (RQ2–4). Table 4 presents the model findings of Model 1 with the auto-regressive effects and cross-sectional correlations for descriptive norms.

**Table 4:** Standardized Estimates for Model 1 and Model 4 on descriptive and injunctive parental and peer norms and snacking behavior

	DN (Model 1)		DN controlled for IN (Model 4)		IN (Model 4)		IN controlled for DN (Model 4)	
	β	S.E.	β	S.E.	β	S.E.	β	S.E.
Stability paths								
Parental norm T1 → Parental norm T2	.35***	.05	.32***	.05	.17*	.07	.17*	.07
Parental norm T2 → Parental norm T3	.46***	.05	.43***	.05	.40***	.07	.35***	.07
Peer norm T1 → Peer norm T2	.22***	.06	.20***	.06	.27***	.07	.27***	.07
Peer norm T2 → Peer norm T3	.36***	.08	.35***	.08	.47***	.07	.44***	.07
Non-core T1 → Non-core T2	.26***	.04	.27***	.04	.27***	.04	.27***	.04
Non-core T2 → Non-core T3	.27***	.07	.25***	.07	.25***	.07	.25***	.07
Core T1 → Core T2	.30***	.04	.28***	.04	.28***	.04	.28***	.04
Core T2 → Core T3	.24***	.06	.24***	.06	.25***	.06	.24***	.06
Cross-lagged paths								
Parental norm T1 → Non-core T2	–	–	−.04	.06	−.05	.06	−.04	.06
Parental norm T2 → Non-core T3	–	–	−.17	.12	−.21*	.10	−.13	.13
Parental norm T1 → Core T2	–	–	−.00	.06	.16**	.05	.16**	.06
Parental norm T2 → Core T3	–	–	.17	.12	.11	.10	.04	.13
Peer norm T1 → Non-core T2	–	–	.01	.06	.05	.06	.06	.06
Peer norm T2 → Non-core T3	–	–	−.03	.10	.03	.11	.08	.11
Peer norm T1 → Core T2	–	–	.07	.05	−.13*	.06	−.15*	.06
Peer norm T2 → Core T3	–	–	.00	.10	−.08	.13	−.14	.13
Non-core T1 → Parental norm T2	–	–	−.20*	.10	-.19 ms	.11	−.17	.12
Non-core T2 → Parental norm T3	–	–	-.16 ms	.08	.14	.09	.14	.09
Non-core T1 → Peer norm T2	–	–	−.12	.10	−.02	.11	−.02	.11
Non-core T2 → Peer norm T3	–	–	−.12	.09	−.06	.09	−.06	.09
Core T1 → Parental norm T2	–	–	−.07 .	09	−.14	.11	−.13	.12
Core T2 → Parental norm T3	–	–	−.07 .	08	.15	.09	.15	.09
Core T1 → Peer norm T2	–	–	−.05 .	09	.05	.11	.04	.11
Core T2 → Peer norm T3	–	–	−.03 .	09	−.10	.09	−.08	.09
Cross-sectional associations								
Parental norm T1 ↔ Peer norm T1	.44***	.04	.40***	.04	.48***	.03	.43***	.04
Parental norm T2 ↔ Peer norm T2	.50***	.05	.48***	.06	.45***	.06	.42***	.06
Parental norm T3 ↔ Peer norm T3	.35***	.07	.35***	.07	.32***	.06	.33***	.07
Parental norm T1 ↔ Non-core T1	−.24***	.04	−.22***	.04	−.22***	.05	−.19***	.05
Parental norm T2 ↔ Non-core T2	−.17**	.06	−.15*	.06	-.12 ms	.07	−.09	.07
Parental norm T3 ↔ Non-core T3	−.05	.08	.00	.08	.06	.09	.03	.08
Parental norm T1 ↔ Core T1	.18***	.04	.16***	.04	.18***	.04	.17***	.04
Parental norm T2 ↔ Core T2	.13	.06	.11 ms	.06	.10 ms	.06	.12*	.06
Parental norm T3 ↔ Core T3	.08	.07	.05	.07	.01	.07	.03	.07
Peer norm T1 ↔ Non-core T1	−.15***	.04	−.11**	.04	−.12**	.04	-.08 ms	.04
Peer norm T2 ↔ Non-core T2	−.15*	.07	-.12 ms	.07	−.10	.08	−.06	.08
Peer norm T3 ↔ Non-core T3	−.05	.08	−.04	.09	.02	.08	−.03	.09
Peer norm T1 ↔ Core T1	.09*	.04	.06	.04	.04	.04	.01	.04
Peer norm T2 ↔ Core T2	.03	.06	.00	.06	.11	.07	.07	.08
Peer norm T3 ↔ Core T3	.08	.07	.08	.08	.04	.08	.10	.09
Non-core T1 ↔ Core T1	−.79***	.02	−.79***	.02	−.79***	.02	−.79***	.02
Non-core T2 ↔ Core T2	−.66***	.04	−.66***	.04	−.66***	.04	−.66***	.04
Non-core T3 ↔ Core T3	−.74***	.05	−.73***	.05	−.74***	.05	−.74***	.05
Parental DN T1 ↔ Parental IN T1	–	–	−.22***	.04	–	–	.22***	.04
Parental DN T2 ↔ Parental IN T2	–	–	.17**	.06	–	–	.17**	.06
Parental DN T3 ↔ Parental IN T3	–	–	.36***	.07	–	–	.36***	.07
Parental DN T1 ↔ Peer IN T1	–	–	.40***	.04	–	–	.40***	.04
Parental DN T2 ↔ Peer IN T2	–	–	.48***	.06	–	–	.48***	.06
Parental DN T3 ↔ Peer IN T3	–	–	.35***	.07	–	–	.35***	.07
Peer DN T1 ↔ Peer IN T1	–	–	.17***	.04	–	–	.17***	.04
Peer DN T2 ↔ Peer IN T2	–	–	.11 ms	.06	–	–	.11 ms	.06
Peer DN T3 ↔ Peer IN T3	–	–	.30***	.07	–	–	.30***	.07
Covariates								
BMI T1 → Core T1	.02	.04	.02	.04	.02	.04	.02	.04
Age T1 → Core T1	−.11**	.04	−.11**	.04	−.11**	.04	−.11**	.04
Sex T1 → Core T1	.05	.04	.05	.04	.05	.04	.05	.04
BMI T1 → Non-core T1	−.07*	.04	−.07*	.04	−.07*	.04	−.07*	.04
Age T1 → Non-core T1	.12**	.04	.12**	.04	.12**	.04	.12**	.04
Sex T1 → Non-core T1	−.02	.04	−.02	.04	−.02	.04	−.02	.04
BMI T1 → Parental norms T1	.05	.04	.05	.04	−.05	.04	−.05	.05
Age T1 → Parental norms T1	−.11**	.04	−.11**	.04	.04	.04	.04	.04
Sex T1 → Parental norms T1	.08*	.04	.09*	.04	.05	.04	.05	.04
BMI T1 → Peer norms T1	.05	.04	.05	.04	.02	.04	.01	.04
Age T1 → Peer norms T1	−.11**	.04	−.10**	.04	−.03	.04	−.03	.04
Sex T1 → Peer norms T1	.08*	.04	.08*	.04	.06	.04	.07	.04

*ms* marginal significant (*p* < .08), * *p* < .05, ** *p* < .01 *** *p* < .001

The cross-sectional findings show that perceived descriptive parental norms were negatively correlated with non-core food intake on T1 and T2 (*p*-values < .01), but not on T3. Descriptive parental norms and core food intake were positively correlated on T1 only (*p* < .001). Notably, the correlations on T2 and T3 were not significant compared to the bivariate correlations (without missing value imputations) presented in Table 2. Perceived descriptive peer norms were only positively correlated with core food intake on T1 (*p* < .05). Perceived descriptive peer norms were negatively correlated with non-core food intake on T1 and T2 (*p* < .001 and *p* < .05, respectively), but not on T3. In addition, peer norms were positively correlated with descriptive parental norms on T1, T2 and T3 (*p*-values < .001).

#### Injunctive norms

**Fig. 2:**
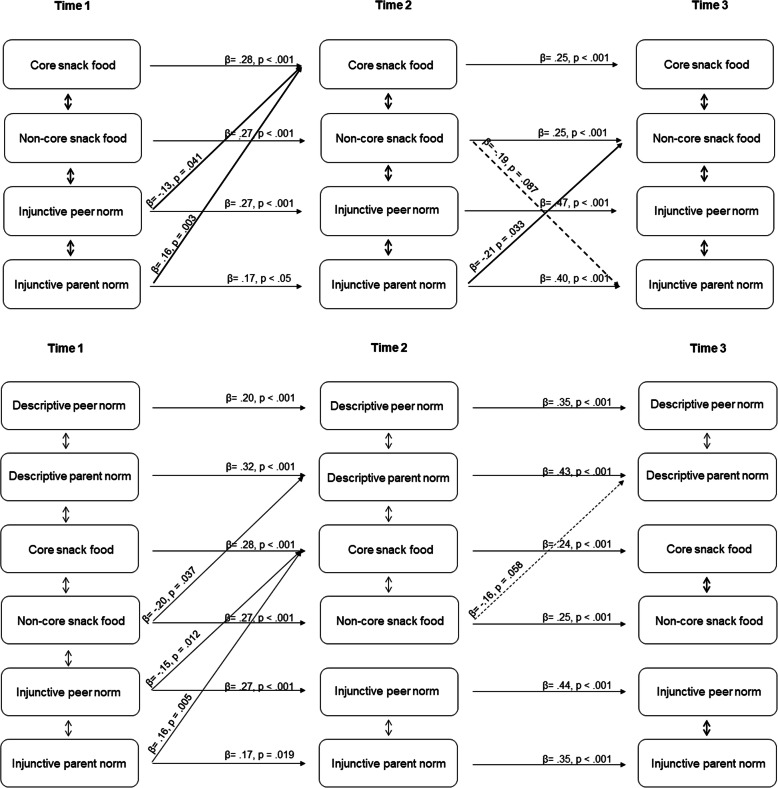
Model 4 for injunctive norms and snacking behavior only (above) and Model 4 for descriptive and injunctive norms in one model (below)

The chi-square difference tests between the models for injunctive norms show that both Model 2 (RQ2 - *forward*) and 4 (RQ4 - *reciprocal*) fit the data better than Model 1 (*p* = .024 and *p* = .032, respectively), and that Model 4 fitted the data better than Model 3 (*p* = .025). Comparing the sample-size adjusted BIC’s between Model 1 and 2 (diff = 11), and Model 1 and 4 (diff = 28), there is stronger evidence that Model 4 provides better insight into the relationship between injunctive norms and snacking behavior, suggesting a bi-directional relationship between injunctive norms and behavior. Figure 2 depicts the findings of Model 4 for injunctive norms (only marginal and statistical significant paths are shown). Table 4 presents also the cross-sectional correlations and control variables.

Figure 2 shows that the injunctive peer norm was negatively associated with core snack intake from T1 to T2 (*p* = .04), but not from T2 to T3 (*p* = .55), and that the parental injunctive norm was positively associated with core snack intake from T1 to T2 (*p* = .003), but not from T2 to T3 (*p* = .30). From T2 to T3, the model shows that there is a significant negative effect of the injunctive parental norm on non-core food intake (β = −.21, *p* = .033) and a marginal reversed effect of non-core food intake on injunctive parental norms (β = −.19, *p* = .087). Non-core food intake was not associated with injunctive peer norms over time. These results suggest that perceived parental pressure to snack healthy can influence snacking behavior of adolescents by affecting core snack intake positively and non-core snack intake negatively over time. In addition, the model provides the first evidence of potential cross-lagged associations between non-core snack food intake and injunctive parental norms. That is, that when adolescents snack unhealthy, they may perceive less pressure of their parents to snack healthy.

The cross-sectional findings resemble the correlations (without missing value imputations) presented in Table 2. They show that injunctive parental norms and core food intake were positively correlated on T1 (*p* < .001) and marginally significant on T2 (*p* = .072). Injunctive peer norms were not correlated with core food intake on T1, T2 and T3. Injunctive parental norms and non-core food intake was only negatively correlated on T1 (*p* < .001) and marginally on T2 (*p* = .064), and injunctive peer norms was also negatively correlated with non-core food intake on T1 only (*p* < .01). In addition, injunctive peer norms were positively correlated with parental injunctive norms on T1, T2 and T3 (*p*-values < .001).

### Additional analyses

As additional analyses, we entered the four norm variables with snacking behavior in one model and compared each of the four models (Model 1 - Model 4) again. All four models provided acceptable fit to the observed data with all Chi-Square test of model fits being significant (*p* < .0001) and according to the values for RMSEA (.034–.037), CFI (.937–.944) and TLI (.897–.905) (see Table 3). The chi-square difference tests between the combined normative models show that Model 4 (RQ4 - *reciprocal*) fits the data better than Model 1 (*p* = .047). Figure 2 depicts the model findings only showing marginal and statistically significant paths without control variables and cross-sectional correlations, which can be found in Table 4.

Compared to our main analyses (Model 4) testing injunctive norms and snacking behavior only, the combined model depicted in Fig. 2 shows similar findings from T1 to T2; however, the cross-lagged paths disappear from T2 to T3 (all *p*-values > .10). Interestingly, this model shows *reversed* paths of non-core snack food intake on parental descriptive norms from T1 to T2 and T2 to T3 (β = −.20, *p* = .037 and β = −.16, *p* = .058, respectively). This suggests that adolescents with higher non-core snack food intake report that they do not see their parents consume core snack foods often.

The cross-sectional findings on T1, T2 and T3 are similar to the two separately tested models for descriptive (Model 1) and injunctive norms (Model 4). In line with the correlations in Table 2, there were strong cross-sectional correlations between parental and peer injunctive and descriptive norms (all *p*-values < .05).

## Discussion

The present study examined the temporal sequence between social norms and snack food intake among adolescents by applying cross-lagged autoregressive models to a three-wave longitudinal study. We provide the first evidence that there are bi-directional relationships between norms and behavior. Our findings from the main and additional analyses suggested that behavior may also affect the perception of norms (*reversed* direction). That is, higher unhealthy snack food intake was negatively associated with the perception of descriptive and injunctive parental norms 1 year later. Further, our study showed that perceived injunctive parental norms were positively associated with healthy snack food intake and negatively associated with unhealthy snack intake (*forward* direction). Injunctive peer norms were negatively associated with healthy snack food intake. In addition, we found no clear evidence that peer norms could be more important compared to parental norms considering (changes in) snacking behavior.

Most experimental research on social norms and eating behavior seem in favor of using descriptive norms to influence eating behavior because they are found to have greater effect on eating behavior compared to injunctive norms, and may avoid potential reactance effects [48–50]. Although we did not manipulate the perceived norms in our study, our findings are important for this field of research when aiming to develop interventions with long term impact [10, 19, 40]. In our study, we did not find an effect over time of perceived peer and parental descriptive norms on (un)healthy snack intake. With regard to peer descriptive norms, this is in line with the only study on longitudinal effects of descriptive norms on snacking behavior among students [35]. Interestingly, the findings from our additional analyses did show a relatively stable reversed effect of unhealthy snacking behavior on the perception of the descriptive parental norm over time; that is, adolescents reporting a relatively higher unhealthy snack intake, reported that they had seen their parents eating healthy snack foods less often in the subsequent years. This may imply that giving the right example as a parent does not necessarily impact their children’s healthy snacking behavior (i.e., modeling); instead, children may use their parents’ unhealthy behavior as an excuse or justification to snack unhealthy. This adds an interesting new dimension to role modeling. Instead of *seeing is doing*, these findings may suggest that *doing is preventing*. So, giving the right example as a parent could prevent their children from snacking unhealthy foods as opposed to stimulating children to consume healthy products. Nevertheless, this assumption is based on the adolescents’ reports only. More longitudinal research is needed on parental (un)healthy snacking behavior by also assessing parents’ snack intake together with their children’s snack intake.

With regard to perceived injunctive norms, this study showed that the peer injunctive norm was negatively associated with healthy snack intake 1 year later, which is in line with previous studies that refer to reactance [32, 33]. In contrast, when adolescents perceived pressure from their parents to snack healthy, they reported to eat relatively more healthy snacks and less unhealthy snacks. Similar to our findings on descriptive norms, we also did not find evidence on injunctive norms that peers would have more influence on snacking behavior than parents over time [16, 29]. These findings are supported by a study examining the importance of different sources of influence among adolescents, in which participants reported that their parents required them to eat healthy foods more often than their friends, teachers and promotion materials from health authorities [51]. Parents were even perceived as being the most effective source in encouraging them to eat healthy foods [51]. Nevertheless, we should be cautious in giving meaning to these findings as they were not consistent across each time point. Additionally, research on parental feeding styles shows that parental pressure to eat could lead to unhealthy eating practices and discouragement of healthy food intake [52, 53]. It may be that the parent injunctive norm taps into a different kind of pressure than parental feeding styles that are measured, for example, by the Child Feeding Questionnaire (CFQ) [54]. Thus other forms of long-lasting interpersonal influence than social norms may play a more prominent role or interact with norms in explaining changes in snacking behavior during this developmental period. Future research should assess other types of social influences and forms of encouragement such as social support, encouragement or sabotage. In addition, it would be interesting to investigate by use of qualitative research what these type of behaviors or influences are and how adolescents know what their parents and peers think they should do.

In our main analyses testing injunctive norms only, we found a cross-lagged pattern between injunctive parental norms and adolescents reporting to have a relatively lower unhealthy snack intake, and in turn, they reported to perceive less parental pressure a year later. Nevertheless, these effects were not apparent anymore when including descriptive norms. Further, a reversed effect of unhealthy snacking on the descriptive parent norm was found in the additional analyses. Although descriptive and injunctive norms are typically treated as independent predictors of behavior, we suggest that the two type of norms are not mutually exclusive and may interact with each other [55]. Our study supports this notion because the descriptive and injunctive norms were highly correlated. It could imply, for example, that injunctive norms may surpass the influence of descriptive norms because adolescents could have used the descriptive norms as indicator of injunctive norms. In addition, most people know others approve of eating fruit and vegetables even if they or others do not eat healthy themselves. Future research should address the possible interaction between these type of norms and researchers are encouraged to investigate the use of social norms in combined designs to advance the research field on social norms and food intake. For example, interventions that make use of peer behaviors to influence a targeted behavior (such as social network interventions) may benefit from including elements tapping into the use of injunctive norms while this behavior could be perceived as a descriptive norm. In this way, it does not elicit a reactance effect on injunctive norms.

Our study had limitations that should be acknowledged. First, the data relies on self-reported recall of snack food intake and perceptions of social norms which may raise concerns about social desirable answers and participation bias. We tried to reduce potential problems related to these concerns by stressing the anonymous data handling to caregivers and participants, and data were assessed during multiple time points. Second, the accuracy of adolescents’ self-report and how they perceive the snacking behavior of their parents may have changed over the years. Third, the precision of our snacking measures and norms could be improved upon. Future research could profit from assessing eating behavior from multiple sources (e.g., by including parent reports). In addition, we assessed norms for parents and peers in general while it is likely that opinions, norms and eating behaviors differ between parents and various friends. Likewise, future research could make a distinction in whether the snack measures are assessed during weekdays or weekend days when adolescents spend more time with their peers or parents. We also used single items to measure social norms on healthy food intake to not overburden the participants in the *MyMovez* project. Future studies are advised to use multiple item measures to improve study reliability, and to assess social norms on unhealthy foods as predictors of snacking (un)healthy foods. Investigating the combination of both healthy and unhealthy social norm perceptions would improve insights in social influences on eating behavior. Fourth, majority of the study sample consisted of non-overweight and few overweight or obese participants. It would be interesting to examine a larger sample of overweight or obese participants to investigate their norm perceptions and snacking behaviors.

In conclusion, this was the first study that investigated the directional nature of the relations between descriptive and injunctive parent and peer norms and snack food intake, which has important theoretical and practical implications. First, we suggest that future social norm studies take into account the bi-directional relationship between norms and behavior in their design and when interpreting their findings. Norms do not only influence our eating behavior, but our eating behavior also influences how we perceive norms. Second, we found that parents expecting their children to snack healthy had a positive influence on healthy snacking behavior whereas only acting as a healthy role model did not. We also suggest that previous recommendations on the use of the descriptive norm type for (long-term) behavior change should be tempered, because our study showed that descriptive and injunctive norms are highly correlated and injunctive norms seem to have a stronger impact than descriptive norms. More research is needed to unravel whether people really perceive descriptive norms as intended or whether they may use it as an indicator of injunctive norms, or vice versa. We also emphasize that further longitudinal work is needed to replicate our findings. The development of effective strategies to improve young people’s eating behaviors requires a better understanding of how social connections influence their eating behavior and vice versa.

## Data Availability

Data and materials of the *MyMovez* project are available in the repository of the Radboud University. The specific datasets used and/or analysed during the current study are available from the corresponding author on reasonable request.

## Footnotes

1 T3 also served as a baseline measurement for the MyMovez intervention at the end of the project during Phase II. In order to schedule all required questionnaires without overburdening the participants, the FFQ was assessed once.

2 Three members of the MyMovez team independently scored kilo caloric values for each of the food items. Inter-coder reliability was consistently high (ĸ = .97).
